# CircFAM114A2 inhibits the progression of hepatocellular carcinoma via miR‐630/HHIP axis

**DOI:** 10.1002/cam4.5894

**Published:** 2023-04-11

**Authors:** Mingshuang Lai, Deyuan Li, Meiliang Liu, Ruirui Zhang, Lijun Wang, Wenyi Peng, Haotian Xu, Siqian Wu, Si Liang, Ye Gu, Aruo Nan, Xiaoyun Zeng

**Affiliations:** ^1^ Department of Epidemiology and Health Statistics, School of Public Health Guangxi Medical University 530021 Nanning Guangxi China; ^2^ Guangxi Colleges and Universities Key Laboratory of Prevention and Control of Highly Prevalent Diseases Guangxi Medical University 530021 Nanning Guangxi China; ^3^ Key Laboratory of Early Prevention and Treatment for Regional High Frequency Tumor (Guangxi Medical University), Ministry of Education Nanning Guangxi China

**Keywords:** circFAM114A2, hepatocellular carcinoma, HHIP, miR‐630

## Abstract

**Background:**

Many studies have shown that circular RNAs (circRNAs) are abnormally expressed in various tumor tissues and served as a key regulator in the occurrence and development of cancer. However, in hepatocellular carcinoma (HCC), the molecular mechanism of circRNAs in body fluids remains to be further explored.

**Methods:**

The expression levels of genes and proteins were detected by quantitative real‐time polymerase chain reaction (qRT‐PCR) and western blotting, respectively. Cell counting Kit‐8 (CCK‐8), 5‐Ethynyl‐2'‐deoxyuridine (EdU), wound healing assay, Transwell assays, flow cytometry, and tumor formation models in nude mice were conducted to investigate the effects of circFAM114A2 on HCC cells both in vitro and in vivo. RNA antisense purification (RAP), dual luciferase reporter assays and rescue assays were carried out to verify the interaction between circFAM114A2, miR‐630 and HHIP.

**Results:**

CircFAM114A2 was significantly downregulated in HCC tissues and was associated with microvascular invasion and lymph node metastasis of HCC patients. We also observed that circFAM114A2 was lowly expressed in HCC plasma, which may serve as an effective biomarker to screen HCC patients from healthy controls (area under curve (AUC)=0.922). In vitro, circFAM114A2 overexpression significantly blunted HCC cell proliferation, migration, invasion, and promoted apoptosis, whereas circFAM114A2 silencing posed opposite effects. In vivo, circFAM114A2 overexpression inhibited the growth of HCC cells. Mechanistically, circFAM114A2 could increase the expression of the tumor suppressor HHIP via acting as a sponge for miR‐630.

**Conclusions:**

CircFAM114A2 exerts a tumor suppressor role in HCC through miR‐630/HHIP axis, and may be served as a potential diagnostic and therapeutic biomarker for HCC patients.

## Graphical abstract

CircFAM114A2 exerts a tumor suppressor role in hepatocellular carcinoma (HCC) through miR‐630/HHIP axis and may be served as a potential diagnostic and therapeutic biomarker for HCC patients.

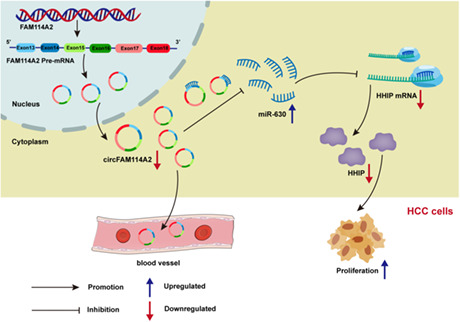

## 1 INTRODUCTION

Hepatocellular carcinoma (HCC) is the most common type of primary liver cancers, ranking as the sixth most common cancer and the third leading cause of cancer deaths worldwide.^1^ Previous studies confirmed that the primary risk factors for HCC are infection with hepatitis B virus or hepatitis C virus, aflatoxin, and alcoholism.^2^ Clinically, approximately 80% of HCC patients are initially discovered in the advanced stages, missing the valuable treatment window for optimal prognosis.^3^, ^4^ Statistics show that the 5‐year overall survival rate of HCC patients is <20%, partly due to a lack of early diagnosis.^5^ As of now, alpha‐fetoprotein (AFP) is the most employed clinical biomarker for the HCC diagnosis; however, the diagnostic sensitivity of this biomarker remains relatively low, at approximately 60%.^6^ Additional biomarkers, such as des‐γ carboxyprothrombin and the L3 fraction of AFP, did not improve the early diagnosis of HCC due to low sensitivity and specificity as well as the fact that they are frequently linked to advanced clinical symptoms of HCC.^7^, ^8^ As a result, there is a critical need to explore more efficient and effective biomarkers to diagnosis HCC at an early stage. In addition, elucidating the molecular mechanisms underlying the development of HCC is of crucial importance for specifying cancer‐intervention strategies.

Circular RNAs (circRNAs) is a family of noncoding RNA, with a covalently closed loop structure generated by the reverse splicing of precursor mRNA.^9^ CircRNAs are circular transcripts, which differ from linear RNAs in that they lack 5′ caps and 3′ polyadenylated tails.^10^ CircRNAs are unique with high stability due to their circular characteristic.^11^, ^12^ Initially, circRNA was considered as an aberrant splicing byproduct with no regulatory function.^13^ Previously, circRNAs have been demonstrated to be characterized by cell, tissue, and developmental stage‐specific expressions, and an increasing number of circRNAs may have been linked to a variety of malignancies.^14^, ^15^ Recently, a growing body of research indicates that circRNAs are critical for many biological processes in various cancer types.^16^ For example, circEYA3 promotes pancreatic cancer progression through sponging miR‐1294, leading to an increase in the expression of c‐Myc and energy production.^17^ CircRNA circDLC1 inhibits HCC progression by interacting with HuR, which can reduce the stability of MMP1 to inhibit the expression of MMP1.^18^ Circ‐EIF6 promotes TNBC progression by encoding EIF6‐224AA, which can interact with MYH9 to activate the Wnt/beta‐catenin pathway.^19^ Although previous studies have shown that many circRNAs are involved in cancer pathophysiology, more investigation is warranted to further ascertain the diagnostic potential, biological function, and mechanism of circulating circRNAs in HCC.

In this study, RNA‐sequencing was used to identify differentially expressed circRNAs in HCC tissues. We found that circFAM114A2 expression was markedly downregulated in HCC tissues and plasma. We hypothesize that circFAM114A2 inhibits HCC progression through competing endogenous RNAs (ceRNA) mechanism. We demonstrated through in vitro and in vivo experiments that circFAM114A2 can inhibit the progression of HCC via miR‐630/HHIP axis.

## 2 MATERIALS AND METHODS

### 2.1 Tissue and plasma samples

From 2018 to 2022, 89 pairs of HCC and adjacent tissues were obtained from the First Affiliated Hospital of Guangxi Medical University and the Affiliated Cancer Hospital of Guangxi Medical University (adjacent tissue refers to liver tissue ≥2 cm away from the cancer tissue, and no cancer cells were observed microscopically). New cases of HCC patients who had not received preoperative radiotherapy, chemotherapy, and radiofrequency ablation were included in our study. All tissue specimens were collected into cryopreserved tubes within half an hour of separation and kept in liquid nitrogen until use. Plasma samples from HCC patients were also obtained from the above two hospitals, while plasma samples from healthy controls were collected from the Physical Examination Department of the First Affiliated Hospital of Guangxi Medical University. The healthy controls were collected from the medical examination population with a frequency that matched HCC patients by age and gender. The healthy subjects with a history of tumor, whether past or present, were excluded from our study. All study participants gave their informed consent, and the Guangxi Medical University Ethics Committee authorized the study.

### 2.2 Cell culture

Human HCC cell lines Huh7, MHCC97H, MHCC97L, HCCLM3, HepG2, Hep3B, and human embryonic kidney cells (293 T) were purchased from the American Type Culture Collection (ATCC). MHCC97H, MHCC97L, HCCLM3, HepG2, Huh7, and 293 T cell lines were cultured in DMEM medium (Gibco, CP21110161839) containing 10% fetal bovine serum (FBS) (Gibco, 10099141C) and 1% penicillin–streptomycin solution (Gibco, 15140112). The MEM medium (Gibco, 8122107) containing 10% FBS and 1% penicillin–streptomycin solution was used to culture Hep3B cells. All cell lines were cultured in a humid environment (5% CO2, 37°C).

### 2.3 Plasmid construction and transfection

CircFAM114A2 overexpression plasmid was constructed by using pcDNA3.1, and the sequence of plasmid was confirmed by sequencing analysis. The extraction of circFAM114A2 plasmid was carried out using a plasmid midi kit (Qiagen, 12,145). The transfection of plasmids was conducted using Lipofectamine™3000 (Invitrogen, L3000015). Additionally, to silence the expression of circFAM114A2 and HHIP, two small interfering RNAs (siRNA) were synthesized by RiboBio (China). The transfection of siRNAs, miR‐630 mimic, and scramble controls were performed using the riboFECT CP Transfection Kit (Ribobio, C10511‐05). All siRNA sequences are shown in Table S1.

### 2.4 RNA extraction, reverse transcription, and quantitative real‐time PCR (qRT‐PCR)

All tissue, cell, and plasma RNAs were extracted with TRIzol™ reagent (Invitrogen, 15,596,018), and the concentration of RNAs were measured with a Nanodrop one detection system (Thermo Fisher Scientific, ND‐ONEC‐W). GoScript™ Reverse Transcription System (Promega, A5002) was conducted reverse transcription of circRNAs and mRNAs. Using the Mir‐XTM miRNA First Strand Synthesis Kit (Takara, 638,315), miRNAs were reverse transcribed. QuantStudio7 (Q7) real‐time‐PCR System was carried out for quantitative real‐time PCR analysis. For miRNA quantification, U6 was applied as an internal reference, and GAPDH and β‐actin were applied as quantitative internal references for mRNA and circRNA. The sequences of all primers are shown in Table S1.

### 2.5 RNase R and Actinomycin D treatment

For RNase R experiment, 1 μg RNA were incubated with 0.15 μL of 3 U/μg RNase R for 10 min at 37°C. Then reverse transcription is performed with RNase R‐treated and control Mock‐treated RNA. Finally, the expression of circFAM114A2 and linear FAM114A2 were detected by qRT‐PCR. For Act‐D experiment, 2 μg/mL Actinomycin D were added to the cells, and the RNAs in each well were taken at 0, 4, 8, 12, and 24 h. Finally, qRT‐PCR was used to detect circFAM114A2 and FAM114A2 expression.

### 2.6 Cell viability analysis

The Cell Counting Kit‐8 (CCK‐8) assay was utilized to examine cell viability. Briefly, in 96‐well plates, 5 × 10^3^ cells were planted. After transfection for 48 h, adding 10 μl of CCK‐8 solution (Dojindo, Japan, CK04) to each well and allowing them to incubate at 37°C for 2 h in the dark. A microplate reader was then used to measure the absorbance of each well at 450 nm.

### 2.7 Cell proliferation analysis

Using the Cell‐Light EdU Apollo567 In Vitro Kit (RiboBio, C10310‐1), the 5‐Ethynyl‐2′‐deoxyuridine (EdU) assay was used to measure cell proliferation rate. About 5 × 10^3^ cells were planted in 96‐well plates, 48 h after transfection, a 1:1000 dilution of EdU solution in cell complete medium was applied and cultivated overnight. Subsequent experiments were carried out according to the manufacturer's instructions. With the use of an EVOS microscope, pictures of the cells were obtained. The proliferative rate in each group was calculated by using Image J software.

### 2.8 Wound healing assay

In 6‐well plates, about 25 × 10^4^ cells were seeded. Transfected cells in each well were scratched vertically with a 200 μL pipetted tip until cell confluence reached 95%. The cells were cultured in fresh complete medium after being washed with PBS three times. The scratch lines were then photographed using a microscope at 0 and 48 h later. Image J was used to compute the cells' relative migration rate.

### 2.9 Apoptotic assay

We used the Apoptosis Detection Kit to detect the cell apoptosis (KeyGEN BioTECH, KGA107). About 3 × 10^5^ cells were seeded in 6‐well plates, after transfection of cells for 48 h, the culture supernatant was collected, then the EDTA‐free trypsin (Solarbio, T1350) was used to digestive cells. Following a precooled PBS wash, the collected cells were gently resuspended in 350 μL of binding buffer. For staining, add 5 μL of FITC and 5 μL of PI in sequence and incubated in the dark for 15 min and 5 min, respectively. Finally, 150 μL of binding buffer was added to the cells prior to flow cytometry testing (Beckman Coulter).

### 2.10 Cell migration and invasion assays

To measure cell invasion, Transwell chambers coated with diluted Matrigel were employed. For cell migration tests, Transwell chambers without diluted Matrigel were utilized (Corning, USA, 3422). 48 h after transfection, the cells were digested and resuspended in serum‐free medium, the upper chamber received 400 μL of suspension containing 3 × 10^4^ cells, and the lower chamber received 700 μL of complete medium and incubated at 37°C for 24 h. Crystal violet was used to stain the cells for 20 min after fixing them with 4% paraformaldehyde for 20 min. When the Transwell was washed with PBS, the cells that have not migrated to the lower chamber were removed with a cotton swab. Under the EVOS microscope, the number of cells in five randomly chosen sections of the chamber were counted.

### 2.11 Animal studies

Four‐week‐old BALB/male nude mice were acquired from the Guangxi Medical University's Animal Experiment Center. The experimental animals were randomly divided into two groups of 6 nude mice each. We screened for stable overexpression of circFAM114A2 (LV‐circFAM114A2) and its negative control (LV‐NC) MHCC97H cell line by infection with lentivirus. The armpits of nude mice were injected with 3 × 10^6^ LV‐circFAM114A2 and LV‐NC MHCC97H cells. Every 3 days, the tumor's growth was observed and measured. The tumor volume was determined using the formula V = 1/2 (length) (width) (width). After 24 days, nude mice were sacrificed, tumors were removed, and weighed. All the removed tumors were sent to the company for immunohistochemical analysis (Servicebio, China). Indicators for immunohistochemical (IHC) analysis include Ki67 (1:500, Servicebio, GB121142) and HHIP (1:100, Affinity, DF13634). The Animal Ethics Committee of Guangxi Medical University approved this study. All the procedures were conducted in accordance with the National Institutes of Health's guide for the care and use of laboratory animals.

### 2.12 Western blot analysis

We lysed cells and extracted proteins using a lysis buffer composed of 10 mM Tris–HCl, pH 7.4, 1% SDS, and 1 mM Na3VO4. A BCA kit (Thermo Fisher Scientific, 23,227) was used to determine the protein concentration. The proteins were separated by polyacrylamide gel electrophoresis containing sodium dodecyl sulfate and then transferred to polyvinylidene fluoride (PVDF) membranes. The membranes were blocked with 5% skim milk for 1 h at room temperature, and subsequently incubated overnight at 4°C in a shaker using a specific primary antibody. HHIP (1:1000, Affinity, DF13634) and GAPDH (1:5000, Affinity, AF7018) were the primary antibodies employed in this study. After washing three times with TBST, the membranes were incubated with rabbit secondary antibodies at room temperature for 1 h. The Clix S6 system was used for chemiluminescence imaging, and ImageJ software was used to calculate the protein bands' gray values.

### 2.13 Nuclear and cytoplasmic separation

Nuclei and cytoplasm from cells were isolated using the PARISTM kit (Invitrogen, AM1921). Briefly, about 1 × 10^7^ cells were collected for subsequently experiment according to the manufacturer's instructions. The relative expression of circFAM114A2 in the cytoplasm and nuclei were, respectively, detected by qRT‐PCR. β‐actin and U6 were, respectively, used as a cytoplasmic and nuclear control.

### 2.14 Fluorescence in situ hybridization (FISH)

We analyzed the subcellular localization of circFAM114A2 by using a fluorescence in situ hybridization kit (RIBOBIO, C10910). 3 × 10^4^ MHCC97H cells were seeded in 12‐well plate with climbers. When the cells were adherent and morphologically intact, the cells in the climbers were fixed with 4% paraformaldehyde for 1 h at room temperature, then incubated for 10 min with permeabilizing agent (0.5% tritonx‐100 in PBS). After fixation with 1% paraformaldehyde again, the cells were dehydrated in gradient of 70%, 80%, 95%, and 100% ethanol for 5 min in order. The hybridization reaction solution was prepared with the probe at a ratio of 19:1 at 42°C for hybridization in the dark overnight. Cells were washed and stained with DAPI for 8 min. Finally, the subcellular localization of circFAM114A2 was analyzed by cell imaging with an LSM800 confocal microscope (Zeiss). The circFAM114A2 FISH probe sequences are shown in Table S1.

### 2.15 RNA antisense purification (RAP)

A RAP kit (BersinBio, Bes5103) was used to verify RNA–RNA interactions. Firstly, three RAP probes modified with Desthio Biotin at the five end of circFAM114A2 were designed, and the RAP probe sequences are shown in Table S1. Approximately 4 × 10^7^ cells were collected, and 40 mL of PBS containing 1% formaldehyde was given to the cells, they were then given to cross‐link for 10 min at room temperature, followed by the addition of 4 mL of 1.375 M Glycine to neutralize for 5 min at room temperature. To fully lyse cells, add lysis buffer, a protease inhibitor, and an RNase inhibitor to the cells sequentially. The RAP probe was ready in the following ways: 3 min of denaturation at 85°C and then quickly placed at ice for later. Then the prepared probe was added to the experimental group, while the control group did not contain the probe. Then, the circFAM114A2 and RNAs that interacts with it were captured by streptavidin magnetic beads. RNAs were eluted with RNA Elution Buffer and purified with phenol‐chloroform‐isoamyl alcohol. Finally, the enrichment of circFAM114A2 and miRNAs were analyzed by qRT‐PCR.

### 2.16 Dual‐luciferase reporter assay

CircFAM114A2 and HHIP wild‐type (WT) and mutant‐type (MUT) vectors were constructed by Feng hui Biotechnology Co, LTD (China). 35 × 10^4^ 293 T cells were planted in 6‐well plates. After culturing for 12 h, cells were co‐transfection with wild‐type or mutant‐type plasmids, and with miR‐630 mimic or mimic NC, respectively. After 36 h of co‐transfection, the firefly fluorescence intensity and Renilla fluorescence intensity in each group were detected using dual‐luciferase reporter kit (Promega, E1910) according to the manufacturer's instructions.

### 2.17 Statistical analysis

SPSS 24.0 and GraphPad Prism 8.0 were used for statistical analysis. All values were expressed as mean ± standard deviation. Both the chi‐squared test and the student's *t*‐test were used to compare the two groups. The relationship between circFAM114A2 expression and clinicopathological variables in HCC patients using the chi‐squared test and Fisher's exact test. Receiver operating characteristic curve (ROC) was performed to calculate the area under the ROC curve (AUC), as well as diagnostic sensitivity and specificity of plasma circFAM114A2. At least three times each experiment was conducted, and a *p* < 0.05 was considered statistically significant.

## 3 RESULTS

### 3.1 Expression and identification of circFAM114A2 in HCC

**FIGURE 1:**
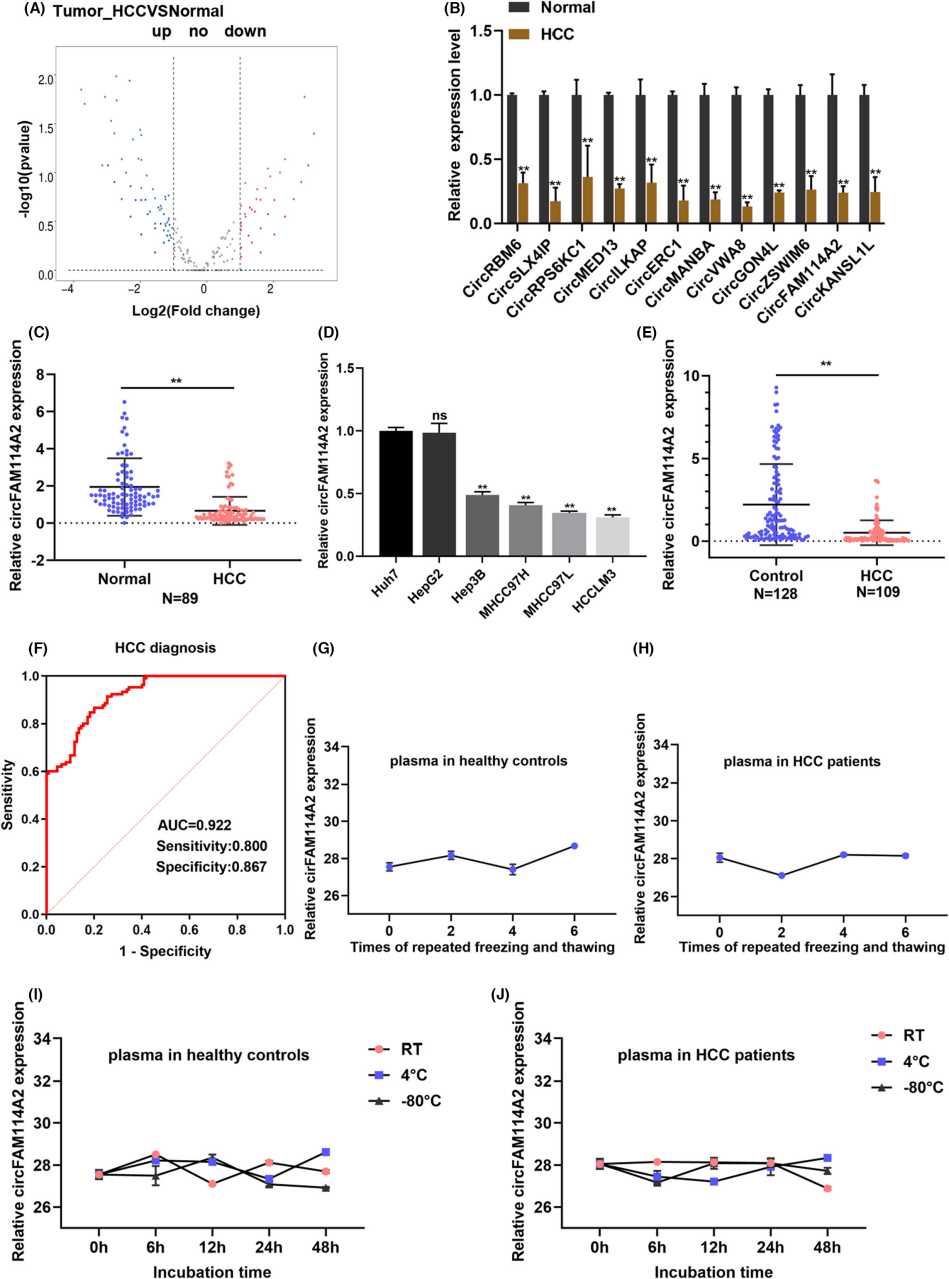
Expression and identification of circFAM11A2 in HCC. (A) Volcanic map of differentially expressed circRNAs between 5 pairs of HCC tissues (B) Relative expression of differentially expressed circRNAs in 30 pairs of mixed HCC tissues and adjacent tissues were detected by qRT‐PCR. (C) Relative expression of circFAM114A2 in HCC tissues. (D) Relative expression of circFAM114A2 in HCC cell lines. (E) Relative expression of circFAM114A2 in plasma of HCC patients and healthy controls. (F) The diagnostic efficacy of plasma circFAM114A2 was evaluated by ROC analysis. (G, H) CT values of plasma ircFAM114A2 in different storage temperature at different time point. (I, J) CT values of plasma circFAM114A2 after the treatment of different times of freeze/thaw. ns indicates no significance; **p < 0.01.

RNA‐sequencing was performed to identify differentially expressed (fold change ≥2.0, *p* < 0.05) circRNAs in HCC (Figure 1A). Subsequently, we selected 12 differentially expressed circRNAs for further validation. qRT‐PCR results showed that among 30 pairs of mixed HCC and adjacent tissue samples, there were 4 circRNAs that were differentially expressed and significantly downregulated, which was consistent with sequencing results (Figure 1B). Then we selected the 4 downregulated circRNAs as candidate circRNAs for further confirmed in 16 pairs of HCC tissues. The results revealed that cicFAM114A2 (circBase ID: hsa_circ_0007773) was the most significantly different among the 4 downregulated circRNAs (*p* < 0.0001) (Figure S1). Based on the validation results above, we selected circFAM114A2 for further investigation. First, we expanded the sample size to further validate the differential expression of circFAM114A2, the results demonstrated that circFAM114A2 was reduced in 89 pairs of HCC tissues (Figure 1C). Next, the relationship between circFAM114A2 expression and patient clinical features were analyzed. The findings demonstrated a connection between circFAM114A2 downregulation and HCC patients' microvascular invasion and lymph node metastases (Table 1). Additionally, we discovered that circFAM114A2 was lower in HCC cell lines (MHCC97H, MHCC97L, and HCCLM3) with high metastatic potential compared to those without metastatic potential (Huh7, HepG2, Hep3B) (Figure 1D). Furthermore, we investigated whether circFAM114A2 is present in body fluids and found that circFAM114A2 was abundant in human plasma. The results from qRT‐PCR detection suggested that circFAM114A2 expression was reduced in HCC plasma as compared to healthy controls (Figure 1E). According to ROC analysis, plasma circFAM114A was an efficient biomarker that could differentiate HCC patients from healthy controls (AUC = 0.922, sensitivity: 0.800, specificity: 0.867, *p* < 0.05) (Figure 1F). Moreover, the stability of plasma circFAM114A2 was investigated, and it was discovered that the expression of circFAM114A2 was mostly unaffected by ambient temperature and repeated freeze–thaw cycles (Figures 1G–J), indicating that circFAM114A2 is expressed in high abundance and stable in plasma.

**TABLE 1:** Association between clinicopathological variables and circFAM114A2 expression in HCC patients

Variables	CircFAM114A2 expression		*p* value
	Low	High	
Age			0.079
≤50	23	19	
>50	15	27	
Gender			0.235
Male	35	39	
Female	3	7	
HBsAg			0.123
Positive	3	12	
Negative	23	33	
Liver cirrhosis			0.803
With	0	2	
Without	26	43	
AFP			0.925
≤400	14	25	
>400	10	17	
Pathological satellite			0.593
Present	5	8	
Absent	33	38	
No. tumor			0.695
Multiple	7	7	
Solitary	31	39	
Tumor size			0.260
>5 cm	25	34	
≤5 cm	3	9	
Microvascular invasion			0.029*
Present	6	1	
Absent	32	43	
Lymphatic metastasis			0.023*
Present	8	2	
Absent	30	42	
BCLC stage			0.178
A	23	22	
B + C	9	17	

* *p* < 0.05.

### 3.2 Circularization structure verification of circFAM114A2

**FIGURE 2:**
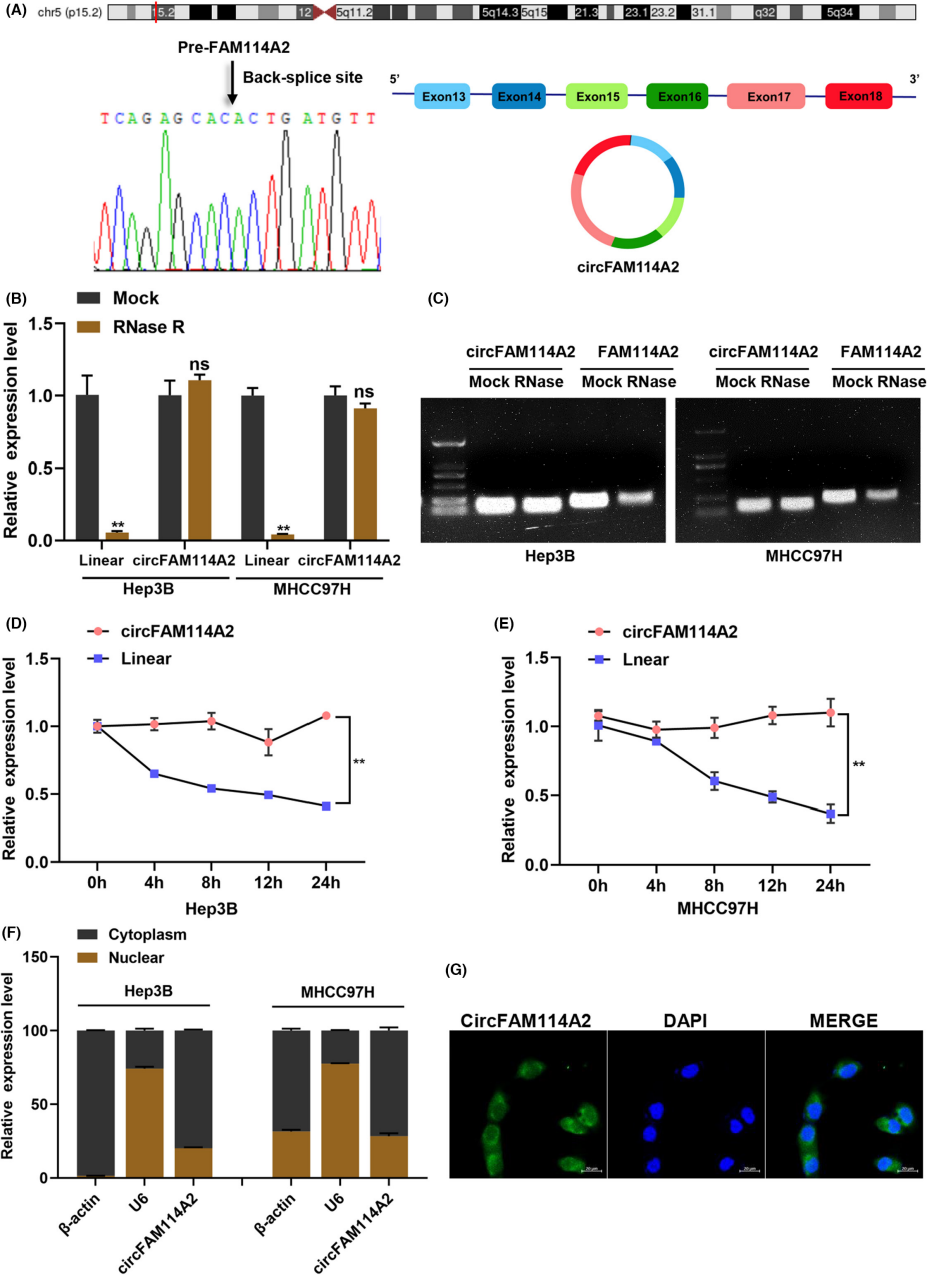
Circularization structure verification of circFAM114A2. (A) Schematic illustration indicating the generation of circFAM114A2 and validated by Sanger sequencing. (B, C) Relative expression of circFAM114A2 and linear FAM114A2 were detected after treated with or without RNase R, and the qRT‐PCR products were performed agarose gel electrophoresis analysis. (D, E) After actinomycin D treatment, relative expression levels of circFAM114A2 and linear FAM114A2 were detected by qRT‐PCR at indicated time point. (F) The cellular distribution of circFAM114A2 was analyzed by nuclear and cytoplasm fractionation assays. β‐Actin and U6 were used as cytoplasmic and nuclear positive controls, respectively. (G) Fluorescence in situ hybridization (FISH) depicting the cytoplasm location of circFAM114A2. Green indicates circFAM114A2. Nuclei were stained with DAPI. Scale bar, 20 μm. ns indicates no significance; **p < 0.01.

According to the annotation of circBase, circFAM114A2 was back spliced from exons 13–18 of the pre‐FAM114A2 with 509 bp. We then conducted several experiments to confirm the circular characteristic of circFAM114A2. Firstly, sanger sequencing was performed with the PCR product of circFAM114A2 to verify the back splicing site of circFAM114A2 (Figure 2A). Then, we observed that circFAM114A2 was substantially more resistant to RNase R than FAM114A2 mRNA when we employed RNase R to digest total RNA from Hep3B and MHCC97H cells (Figure 2B, C). Additionally, we treated cells with the transcription inhibitor actinomycin D, the results demonstrated that circFAM114A2 has a longer half‐life than linear FAM114A2 (Figure 2D,E). Nucleocytoplasmic separation assays showed that circFAM114A2 was predominantly localized in the cytoplasm rather than the nucleus (Figure 2F), where FISH assays confirm further (Figure 2G).

### 3.3 CircFAM114A2 inhibits the malignant phenotypes of HCC cells in vitro

**FIGURE 3:**
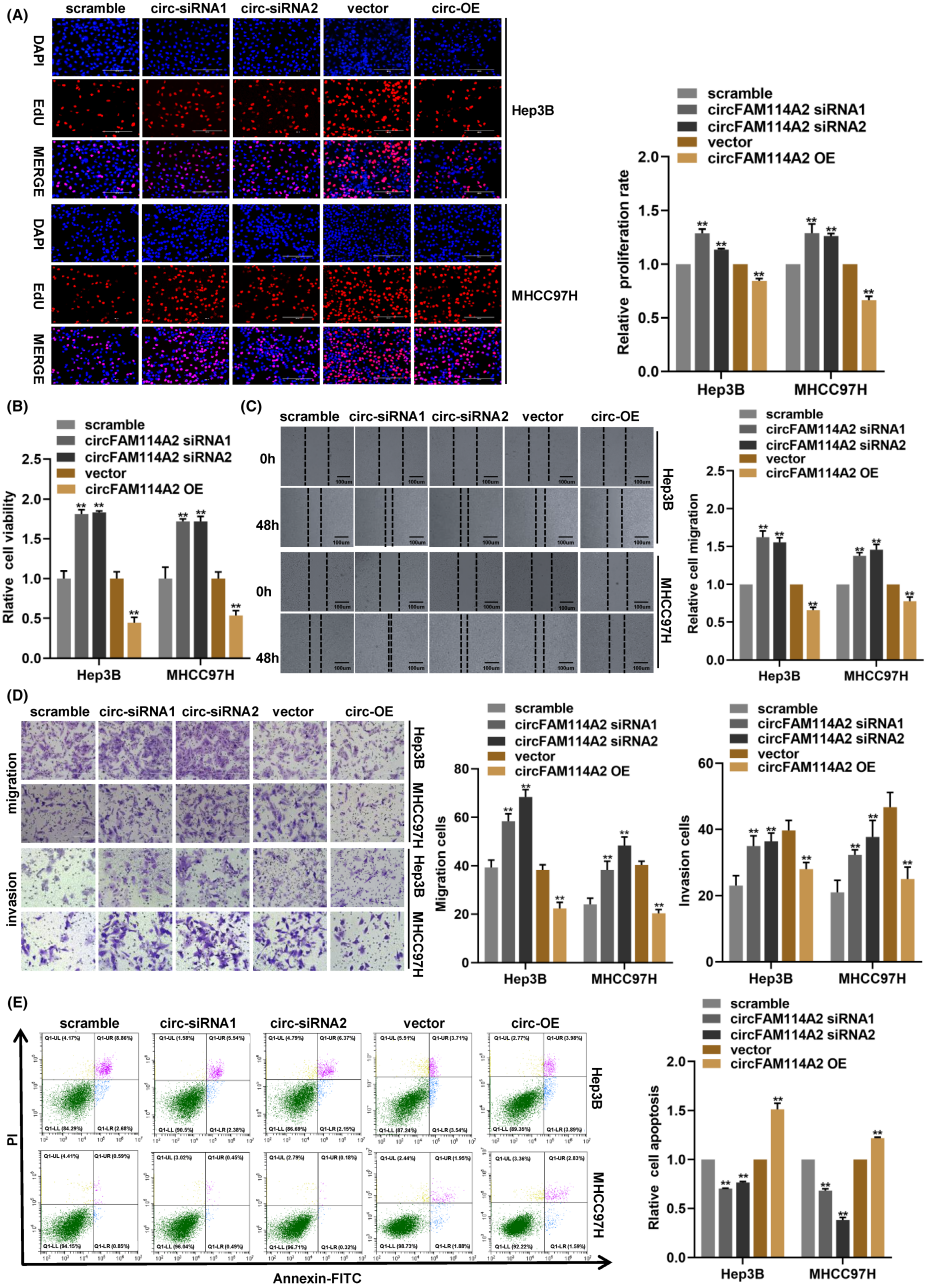
CircFAM114A2 inhibits the malignant phenotypes of HCC cells in vitro. (A) EdU assays were performed to detect cell proliferation when circFAM114A2 was silenced or overexpressed. Scale bars, 200 μm. (B) CCK‐8 assay detected transfected cell viability. (C) Cell migration was evaluated by wound healing assay. (D) Transwell assays were used to test cell migration and invasion. Scale bars, 200 μm. (E) Flow cytometry assay was used to detected cell apoptosis. **p < 0.01.

In order to explore how circFAM114A2 functions biologically in HCC, an overexpression plasmid of circFAM114A2 was constructed and two siRNAs were designed. The silencing and overexpression efficiencies of circFAM114A2 were assessed (Figure S2A). When circFAM114A2 was silenced or overexpressed, the expression of FAM114A2 was unaffected (Figure S2B), indicating that the silencing and overexpression of circFAM114A2 was specific. Subsequently, in vitro cell experiments were carried out to examine how circFAM114A2 affected the HCC cells' malignant phenotypes. The results of the EdU and CCK‐8 experiments demonstrated that circFAM114A2 silencing could increase cell proliferation and viability, whereas overexpression could dramatically decrease both (Figure 3A,B). Both the wound healing assay and the transwell assay were conducted to assess how circFAM114A2 affected cell migration, the results showed that overexpression of circFAM114A2 greatly impaired cell migration capacity, whereas silencing circFAM114A2 facilitated cell migration (Figure 3C,D). Transwell invasion assay suggested that silencing circFAM114A2 could increase the invasive ability of cells, while overexpression showed the opposite results (Figures 3D). We also used flow cytometry to examine how circFAM114A2 affected cell apoptosis, the results demonstrated that overexpression of circFAM114A2 could enhance cell apoptosis while suppressing circFAM114A2 could decrease cell apoptosis (Figure 3E). Collectively, our in vitro experiments revealed that circFAM114A2 suppressed the malignant phenotypes (proliferation, migration, and invasion) of HCC cells.

### 3.4 CircFAM114A2 inhibits the growth of HCC cells in vivo

**FIGURE 4:**
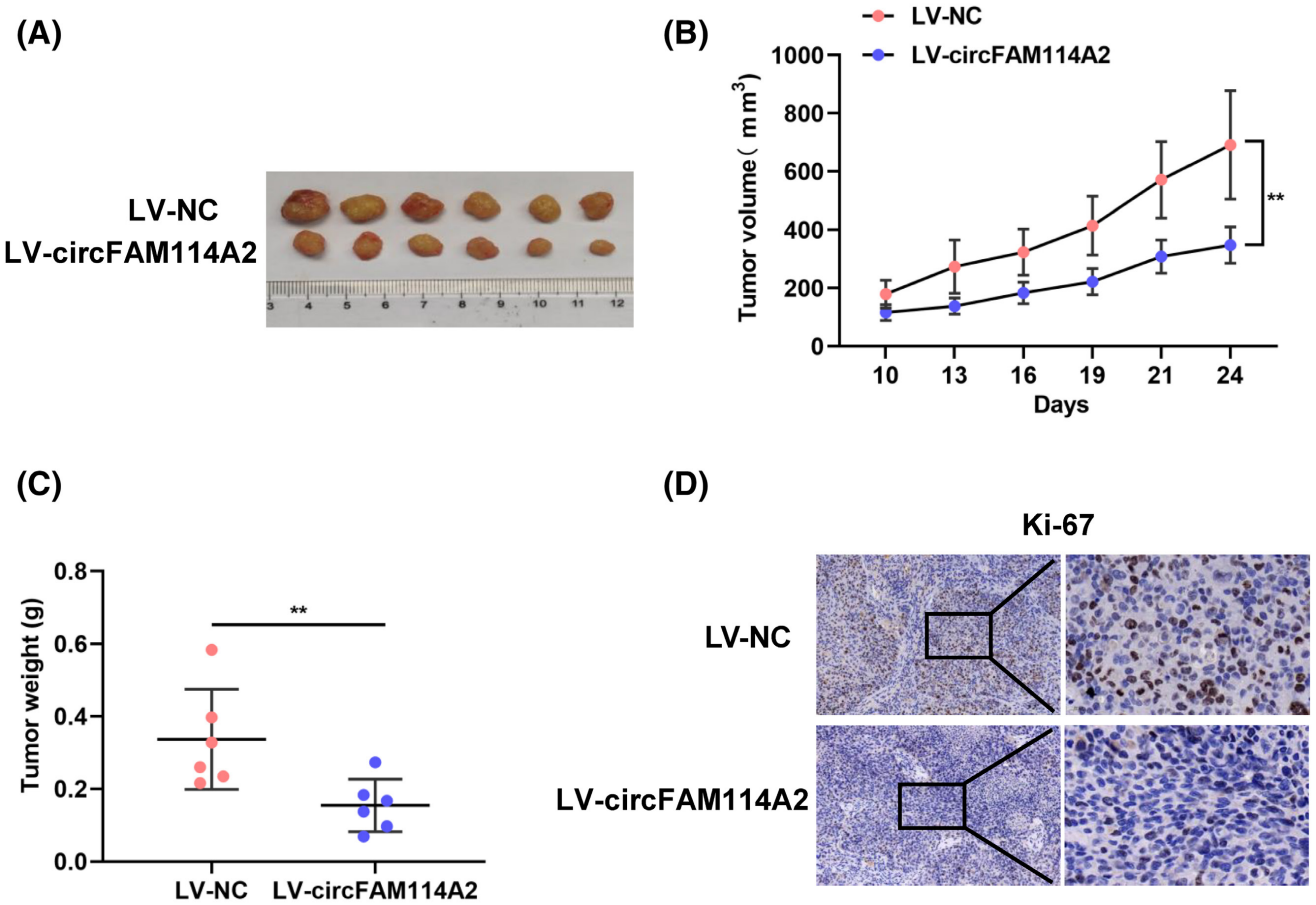
CircFAM114A2 inhibits HCC growth in vivo. (A) Images of subcutaneous xenografts in nude mice. (B) the volume of subcutaneous xenografts in nude mice was measured every three days. (C) All mice were sacrificed, and the xenograft tumors were removed and weighed. (D) IHC analysis of the expression of Ki67. The figures were imaged at 100× and 400× magnification. Scale bar, 100 μm and 20 μm. **p < 0.01.

To learn more about the biological function of circFAM114A2, we established a BALB/C nude mouse xenograft model to conduct an in vivo experiment. Firstly, MHCC97H cells that stably overexpressing circFAM114A2 (LV‐circFAM114A2) or negative control (LV‐NC) were successfully constructed (Figure S2C). The specific overexpression effectiveness of circFAM114A2 in MHCC97H cells was then confirmed by an qRT‐PCR assay, and the results revealed that circFAM114A2 was overexpressed in MHCC97H cells (Figure S2D), while FAM114A2 expression levels were unaffected (Figure S2E). The results from our animal studies showed that, compared with those in the LV‐NC group, the LV‐circFAM114A2 group's tumor growth rate was slower and the tumor weight was smaller (Figures 4A–C). Additional IHC analysis suggested that the Ki67 expression in the LV‐circFAM114A2 group was decreased as compared to the LV‐NC group (Figure 4D). Taken together, circFAM114A2 suppressed the proliferation of HCC cells in vivo.

### 3.5 CircFAM114A2 upregulates HHIP expression via “miR‐630 sponge”

**FIGURE 5:**
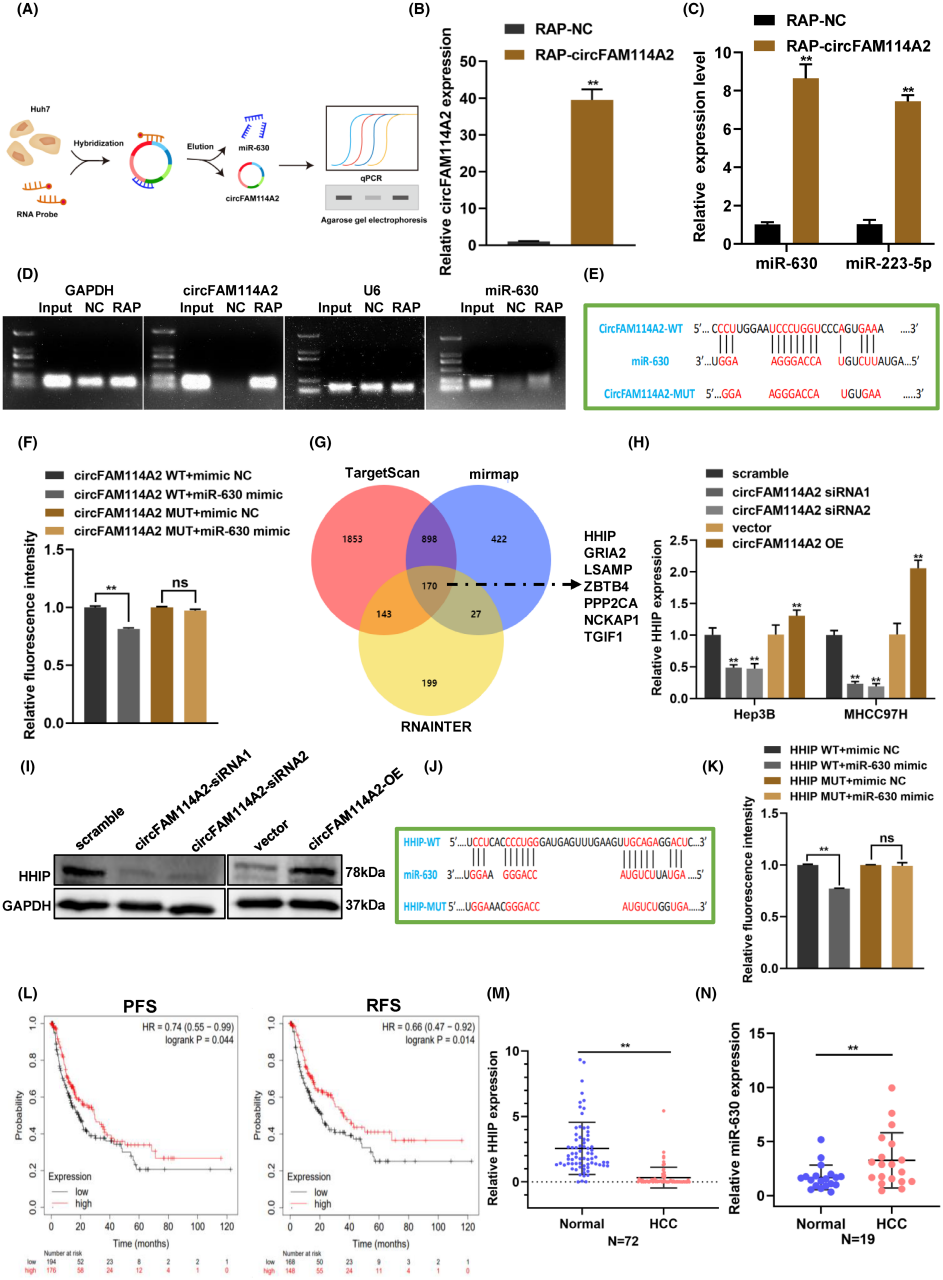
CircFAM114A2 upregulates HHIP expression via “miR‐630 sponge”. (A) RAP experiment's schematic diagram (B–D) Following the RAP experiment, qRT‐PCR was used to determine the enrichment of circFAM114A2 and potential miRNAs. The qRT‐PCR products were then processed to nucleic acid gel electrophoresis using a 1% agarose gel. (E) Schematic diagram of binding sites between miR‐630 and circFAM114A2 or its mutant type (circFAM114A2 MUT). (F) Luciferase reporter assay was performed to validate the interaction between circFAM114A2 and miR‐630. (G) The TargetScan, miRmap and RNA Interactome databases were used to predict miR‐630 targeted mRNAs. (H) Relative expression levels of HHIP was detected in circFAM114A2 silenced and overexpressed cells. (I) Protein level of HHIP was detected by western blot. (J) Schematic diagram of binding sites between miR‐630 and HHIP or its mutant type (HHIP‐MUT). (K) Luciferase reporter assay was performed in 293 T cells to validate the interaction between HHIP and miR‐630. (L) Survival analysis of the expression of HHIP between clinic PFC and DFS. (M) Relative expression of HHIP in HCC tissues. (N) Relative expression of miR‐630 in HCC tissues. **p < 0.01.

As the results shown that circFAM114A2 was mainly distributed in the cytoplasm, we hypothesized that circFAM114A2 may perform its biological role through operating as a “miRNA sponge”. We first applied the Cancer‐Specific CircRNA Database (CSCD) to predict which miRNAs may be bound to circFAM114A2. We found that miR‐630 and miR‐223‐5p may be the possible targets of circFAM114A2 binging. To confirm these binding, we employed RAP experiment to examine the interaction between circFAM114A2 and the two miRNAs (Figure 5A). The RAP results showed that circFAM114A2 specific probe could enrich circFAM114A2 significantly (Figure 5B), as well as miR‐630 and miR‐223‐5p, and miR‐630 has higher enrichment than miR‐223‐5p (Figure 5C). The results following agarose gel electrophoresis further confirmed that miR‐630 has a higher enrichment than that of miR‐223‐5p (Figure 5D). Thus, we selected miR‐630 as the targeted miRNA of circFAM114A2. To further confirm the binding relationship between circFAM114A2 and miR‐630, we constructed wild‐type and mutant vectors of circFAM114A2 using the predicted binding sites between miR‐630 and circFAM114A2 (Figure 5E). We then examined the efficiency of miR‐630 mimic and determined that miR‐630 was overexpressed in HCC cells (Figure S2F). After transfection of miR‐630 mimic, the dual‐luciferase reporter assays were carried out, and the results showed that, in comparison to transfection of mimic NC, the luciferase activity of circFAM114A2‐WT was significantly reduced, while the luciferase activity of circFAM114A2‐MUT did not change (Figure 5F).

We then used TargetScan (https://www.targetscan.org/vert_71/), miRmap (https://mirmap.ezlab.org/app/), and RNA Interactome Database (http://rnainter.org/) databases to further estimate the likely targets of miR‐630, and 170 target genes were found. Among them, 7 target genes that act as tumor suppressors were chosen for further verification based on functional analysis and prior findings (Figure 5G). According to the results of qRT‐PCR, the expression of HHIP mRNA dramatically changed after circFAM114A2 was silenced and overexpressed, respectively (Figure 5H). However, none of the other six candidate genes' expression were impacted. Additionally, western blot examination of the levels of the HHIP protein in samples with silenced and overexpressed circFAM114A2 further supported the results from qRT‐PCR (Figure 5I). Therefore, HHIP was chosen as downstream target genes of miR‐630. To verify the interaction between miR‐630 and HHIP, we constructed wild‐type and mutant vectors of vectors of HHIP based on the binding sites of miR‐630 and HHIP (Figure 5J). The results from dual‐luciferase reporter assay demonstrated that miR‐630 mimic could dramatically lower luciferase activity in the HHIP‐WT group, but not in the HHIP‐MUT group (Figure 5K). Data from The Cancer Genome Atlas (TCGA) revealed that HHIP was considerably downregulated in a variety of cancers, including liver cancer (Figure S3A,B). Furthermore, using survival analysis, it was discovered that the lower expression of HHIP was linked to shorter recurrence‐free survival (RFS) and progression‐free survival (PFS) of HCC patients (Figure 5L). Additional qRT‐PCR analysis revealed that HHIP was markedly downregulated in HCC tissues (Figure 5M), while miR‐630 was upregulated in HCC tissues (Figure 5N). These results suggest that circFAM114A2 bind to miR‐630 to upregulate the expression of the tumor suppressor HHIP indirectly.

### 3.6 CircFAM114A2 suppresses HCC progression through miR‐630/HHIP axis

**FIGURE 6:**
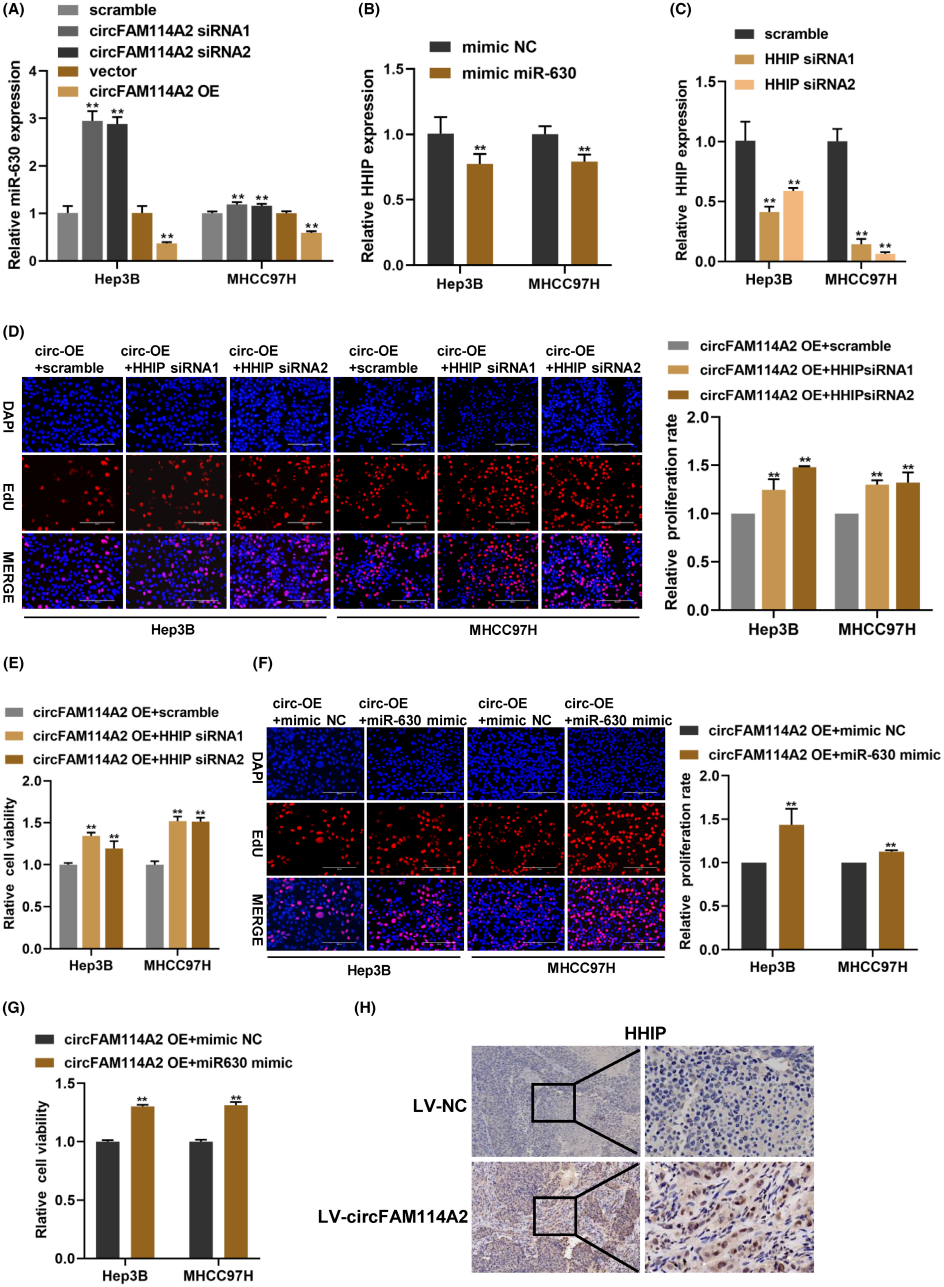
CircFAM114A2 suppresses HCC progression through miR‐630/HHIP axis. (A) Relative expression of miR‐630 were detected by qRT‐PCR in circFAM114A2 silenced or overexpressed cells. (B) Relative expression of HHIP mRNA in miR‐630 overexpressed cells. (C) The silenced efficiency of HHIP was detected by qRT‐PCR. (D) EdU assays were used to evaluate cell proliferation after co‐transfection with circFAM114A2 overexpression plasmid and HHIP siRNA. Scale bars, 200 μm. (E) CCK‐8 assays were used to evaluate cell viability after co‐transfection. (F) EdU assays were used to evaluate cell proliferation after co‐transfection with circFAM114A2 overexpression plasmid and miR‐630 mimic. Scale bars, 200 μm. (G) CCK‐8 assays were used to evaluate cell viability after co‐transfection. (H) IHC analysis of the expression of HHIP. The figures were imaged at 100× and 400× magnification. Scale bar, 100 μm and 20 μm. **p < 0.01.

To further verify the circFAM114A2/miR‐630/HHIP axis, we first measured the expression of miR‐630 after circFAM114A2 was silenced or overexpressed. We found that circFAM114A2 was negatively regulating the expression of miR‐630 (Figure 6A). Additionally, the expression of HHIP mRNA was drastically reduced in Hep3B and MHCC97H cells following transfection with a miR‐630 mimic (Figure 6B). Subsequently, we used two siRNAs to reduce the expression of HHIP, and qRT‐PCR results showed that HHIP mRNA expression was both decreased in Hep3B and MHCC97H cells, respectively (Figure 6C). Then rescue experiments were carried out to further verify the circFAM114A2/miR‐630/HHIP axis. EdU assays showed that silencing HHIP could reverse the inhibition of cell proliferation induced by circFAM114A2 overexpression (Figure 6D). Results from CCK‐8 assays suggested that silencing HHIP could reverse the inhibition of cell viability caused by circFAM114A2 overexpression (Figure 6E). Additionally, reduced cell proliferation and cell viability caused by circFAM114A2 overexpression could be greatly reversed by miR‐630 mimic when compared to mimic NC (Figures 6F,G). According to IHC analyses, tumors from the nude mice displayed higher levels of HHIP expression in the LV‐circFAM114A2 group compared with that in the LV‐NC group (Figure 6H). These findings collectively suggested that circFAM114A2 suppressed the growth of HCC cells via miR‐630/HHIP axis.

## 4 DISCUSSION

In this study, we found for the first time that circFAM114A2 expression was markedly downregulated in HCC tissues and plasma, and the downregulation of circFAM114A2 was correlated with the microvascular invasion and lymphatic metastasis in HCC patients, Additionally, circFAM114A2 inhibited the growth of HCC cells both in vitro and in vivo. Mechanistically, circFAM114A2 overexpression could enhanced the tumor suppressor HHIP via “miR‐630 sponge”. Our study revealed plasma circFAM114A2 as a potent biomarker for HCC, and elucidated the biological functions and regulatory mechanisms of circFAM114A2 in HCC, making it a promising therapeutic target for HCC patients.

CircRNAs are resistant to the digestion of linear digestive enzymes, thus having a greater stability than linear RNAs.^20^ Thus maybe serving as possible biomarkers for diseases.^21^ Numerous research conducted in recent years have demonstrated that circRNAs are stable in human body fluids and can be used as diagnostic biomarkers and prognostic indicators for cancers.^22^ For example, circSPARC was elevated in plasma of colorectal cancer patients and was revealed to be a promising biomarker (AUC = 0.8613).^23^ Exosomal circ‐0051443 was identified as a novel HCC diagnostic marker (AUC = 0.8089).^24^ In the present study, we discovered that circFAM114A2 was highly abundant and stable expression in plasma, suggesting a possible application of circFAM114A2 for HCC early diagnosis.

In recent years, an increasing number of circRNAs are being discovered to be differential expressed in a variety of tumor tissues. These circRNAs were revealed to be participate in a variety of physiological and pathological processes, such as proliferation, metastasis, invasion, and apoptosis.^25^, ^26^, ^27^ In the current study, the biological role of circFAM114A2 in HCC was explored using gain and loss of function assays, and we discovered that silencing circFAM114A2 could enhance cell proliferation, migration, and invasion, while inhibited cell apoptosis. On the contrary, overexpressing circFAM114A2 had the opposite effects observed on silencing circFAM114A2. Furthermore, our in vivo study showed that circFAM114A2 could prevent HCC cells from proliferating. In summary, our in vitro and in vivo experiments indicated that circFAM114A2 play a vital regulatory role in the progression of HCC.

MiRNAs is a type of small noncoding RNA with 18–25 nucleotides and control gene expression at the post‐transcriptional stage.^28^ Numerous studies have shown that circRNAs in the cytoplasm primarily exert their roles by serving as “miRNA sponges,” which regulate the expression of mRNAs by targeting their 3′UTR region.^29^, ^30^ Numerous investigations have demonstrated that “miRNA sponge” is how cytoplasmic circRNAs carry out their biological role.^17^, ^31^ And it was discovered that the majority distribution of circFAM114A2 was present in the cytoplasm. we assume that it has post‐transcriptional regulatory activities. In order to further explore whether circFAM114A2 regulate the progression of HCC through the ceRNA mechanism, we first used bioinformatic analysis to predict the probable miRNAs targeted to circFAM114A2. Then, we used RAP and dual‐luciferase reporter assays to confirm that circFAM114A2 and miR‐630 interact. Previous studies have suggested that miR‐630 is crucial for various tumor types. For instance, miR‐630 is significantly overexpressed in renal cell carcinoma tissues and has been shown to facilitate renal cell carcinoma cell proliferation, migration, invasion, and inhibition of apoptosis.^32^ MiR‐630 targeting KLF6 and promotes the proliferation of epithelial ovarian cancer.^33^ In our study, we showed that miR‐630 was upregulated in HCC tissues. Besides, qRT‐PCR results demonstrated that overexpression and silencing of circFAM114A2 had opposite effects on miR‐630 expression. Additionally, miR‐630 overexpression could lower the expression of its downstream gene HHIP in HCC cells. Moreover, rescue experiments indicated that miR‐630 overexpression could reverse the proliferation‐inhibiting effect of circFAM114A2 overexpression on HCC cells. Collectively, our study demonstrated that miR‐630 functions as an oncogene in HCC, which was in line with earlier research on other cancers.

Hedgehog interacting protein (HHIP) is a key regulator of fundamental processes in embryonic development and an endogenous antagonist of the Hedgehog signaling pathway.^34^, ^35^ Increasing studies evidenced that aberrant activation of the Hedgehog signaling pathway is one of the main causes of cancer progression,^36^, ^37^ especially serving as is a negative regulator of Hedgehog signaling pathway.^35^ Previous research has demonstrated that HHIP acts as a tumor suppressor in a variety of malignancies, such as liver cancer, glioblastoma, and gastric cancer,^38^, ^39^, ^40^ According to bioinformatic analysis, miR‐630 may bind to HHIP, which was verified by dual‐luciferase reporter assay. Additionally, our bioinformatic analysis suggested that HHIP was downregulated in liver cancer and the downregulation of HHIP was associated with the PFS and RFS of HCC patients. Further qRT‐PCR results verified the downregulation of HHIP in HCC tissues. Moreover, rescue studies demonstrated that HHIP silencing could abolish the circFAM114A2 overexpression‐induced proliferation‐inhibiting effect on HCC cells. Therefore, our study confirmed that circFAM114A2 enhances the tumor suppressor HHIP by acting as a sponge for miR‐630, which, in turn, prevents the progression of HCC.

This study also has some limitations. First, the results of the CCK‐8 assay only reflect the cell viability at one time point without using graphs depicting changes over time. Second, the potential mechanism on how circFAM114A2 expression is downregulated in HCC remains to be clarified. Finally, whether plasma circFAM114A2 can be used as a diagnostic marker for HCC needs further validation in a larger patients cohort.

In conclusion, our investigation demonstrated that circFAM114A2 expression was markedly downregulated in HCC tissues and plasma and that this low expression was related to the clinicopathological characteristics of HCC patients. We preliminarily confirmed that circFAM114A2 inhibits the progression of HCC through the circFAM114A2/miR‐630/HHIP axis. Our findings supported that circFAM114A2 may be a promising diagnostic marker and therapeutic target for HCC patients.

## AUTHOR CONTRIBUTIONS

**Mingshaung Lai:** Data curation (lead); formal analysis (lead); validation (lead); visualization (lead); writing – original draft (lead). **Deyuan Li:** Data curation (equal); formal analysis (lead); validation (lead); visualization (lead); writing – original draft (lead). **Meiliang Liu:** Data curation (lead); formal analysis (lead); validation (lead); visualization (lead); writing – original draft (lead). **Ruirui Zhang:** Methodology (lead); supervision (lead); writing – review and editing (equal). **Lijun Wang:** Supervision (equal); writing – review and editing (equal). **Wenyi Peng:** Methodology (equal); supervision (equal). **Haotian Xu:** Methodology (supporting); validation (supporting); visualization (supporting). **Siqian Wu:** Methodology (supporting); validation (equal); visualization (supporting). **Si Liang:** Methodology (supporting); visualization (supporting). **Ye Gu:** Methodology (supporting); visualization (supporting). **Aruo Nan:** Conceptualization (lead); methodology (lead); supervision (lead); writing – review and editing (lead). **Xiaoyun Zeng:** Conceptualization (lead); funding acquisition (lead); resources (lead); supervision (lead); writing – review and editing (lead).

## FUNDING INFORMATION

This work was supported by The Central Government to Guide Local Science and Technology Development Funds, Guangxi, China [Guike ZY22096018], The Key Laboratory of Early Prevention and Treatment for Regional High Frequency Tumor (Guangxi Medical University), Ministry of Education, Guangxi, China [GKE‐ZZ202201], and National Natural Science Foundation of China (No. 82060616).

## CONFLICT OF INTEREST STATEMENT

The authors declare that they have no conflict of interest.

## ETHICS STATEMENT

The animal research was approved by The Guangxi Medical University Laboratory Animal Center. The studies involving human participants were reviewed and approved by The Ethics Committee of Guangxi Medical University. The patients/participants provided their written informed consent to participate in this study.

## Supporting information


Figure S1–S3.

Table S1.

## Data Availability

All data are available upon request.
