# Supplementary material for: CircFAM114A2 inhibits the progression of hepatocellular carcinoma via miR‐630/HHIP axis
Source: Cancer Med. 2023 Apr 11;12(11):12553–68. doi: 10.1002/cam4.5894 (PMC10278467; doi:10.1002/cam4.5894)
Supplement: Supplementary file 1 — Figure S1–S3. [file CAM4-12-12553-s002.pdf]

Figure S1

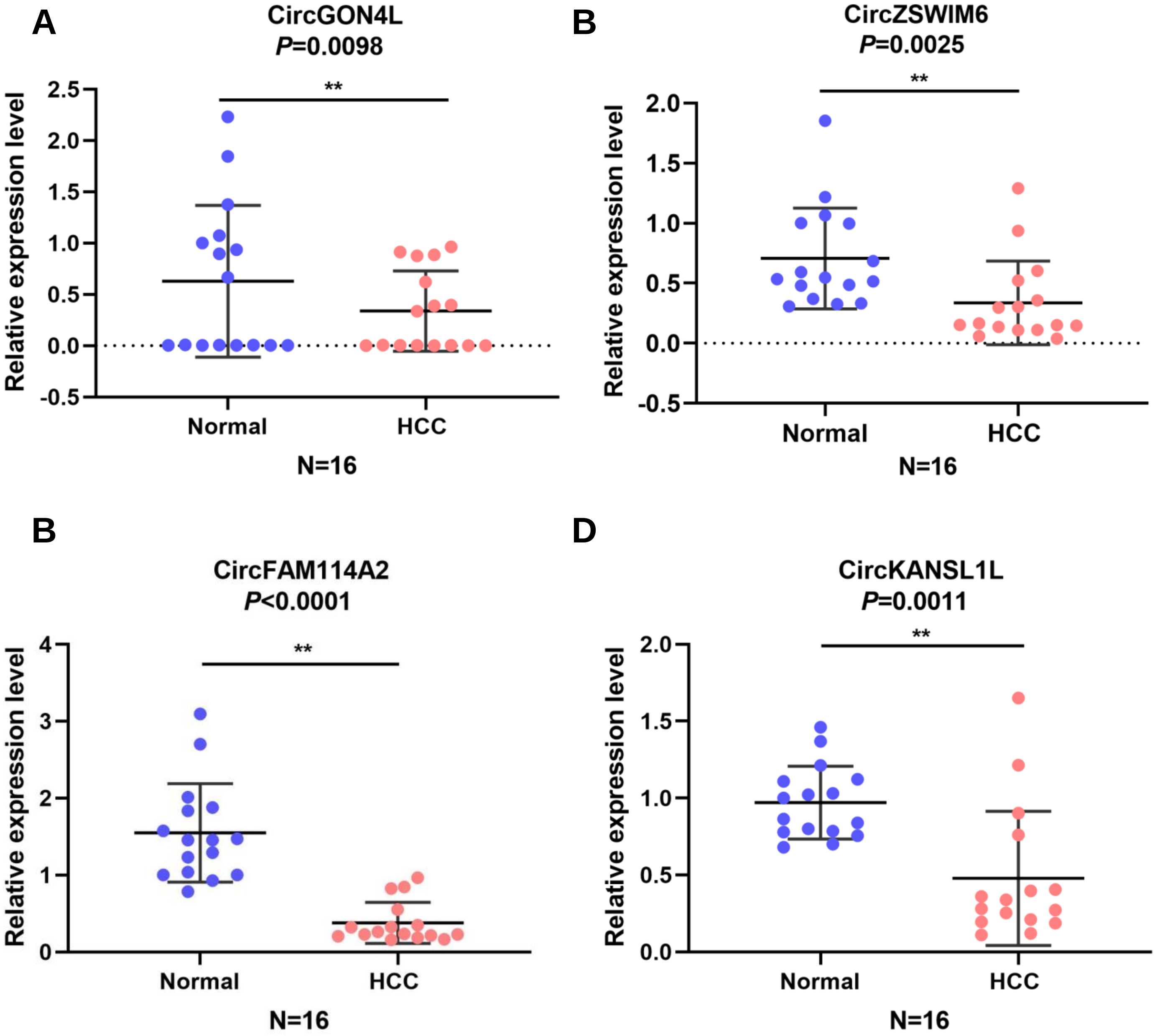

Figure S1. Screening of circFAM114A2. (A-D) Relative expression levels of four candidate circRNAs in 16 pairs of HCC and adjacent tissues were detected by qRT-PCR. ** $p < 0.01$.

Figure S2

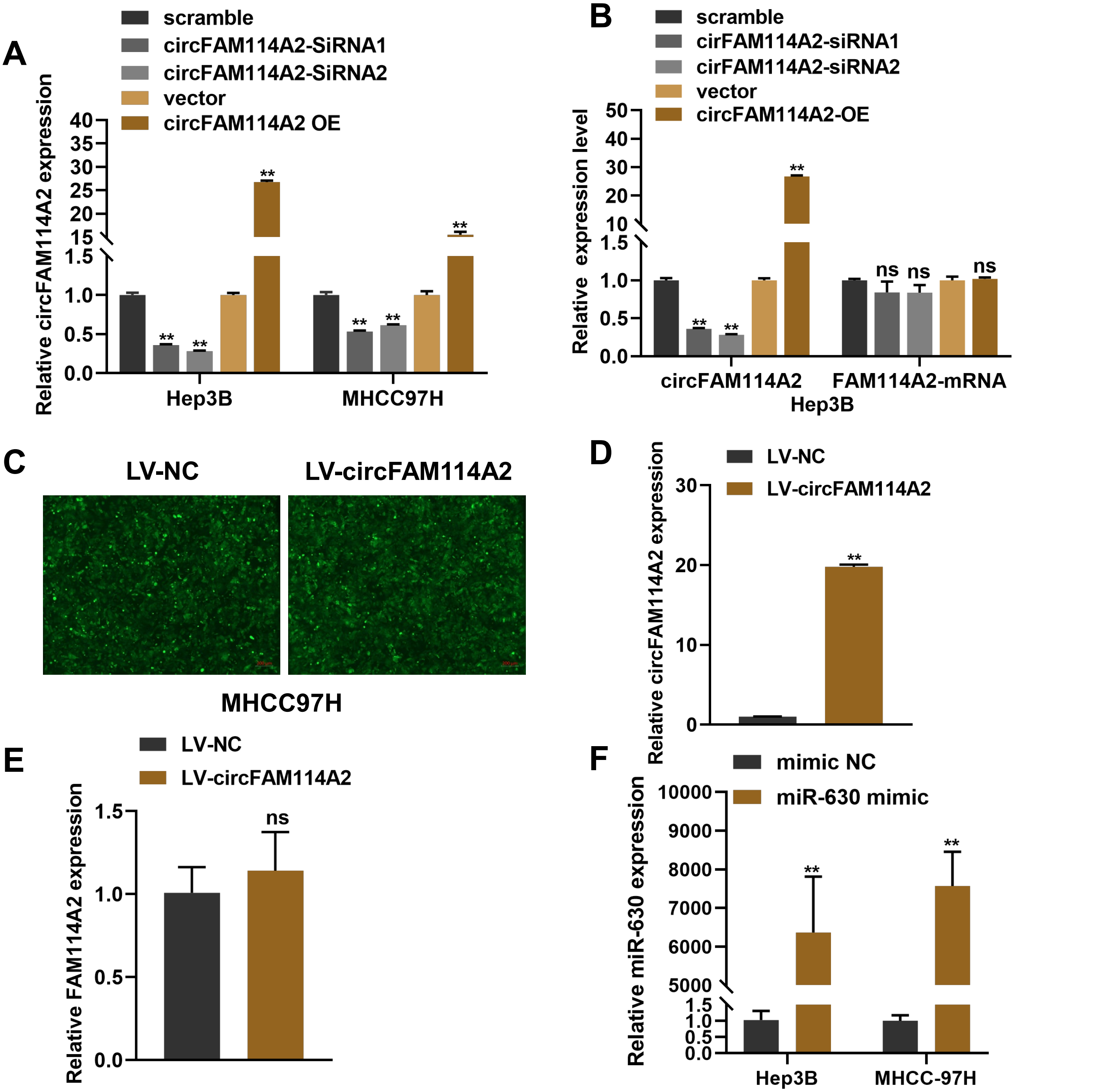

Figure S2. Transfection efficiencies of circFAM114A2.

(A) The silencing and overexpression efficiency of cicFAM114A2 were determined by qRT-PCR. (**B**) Relative expression of circFAM114A2 and FAM114A2 was detected in circFAM114A2 silenced and overexpressed cells. **(C)** MHCC97H cells were transfected with lentivirus overexpressing circFAM114A2 (LV-circFAM114A2) and negative control (LV-NC), the green fluorescence was observed by fluorescence microscope. Scale bars, 200µm. **(D)** The stably overexpression efficiency of circFAM114A2 was determined by qRT-PCR. **(E)** Relative expression level of FAM114A2 in LV-NC group and LV-circFAM114A2 group were detected by qRT-PCR. **(F)** When cells were transfected with miR-630 mimic or mimic NC, the relative expression level of miR-630 was detected by qRT-PCR. ns indicates no significance, ** $p < 0.01$.

Figure S3

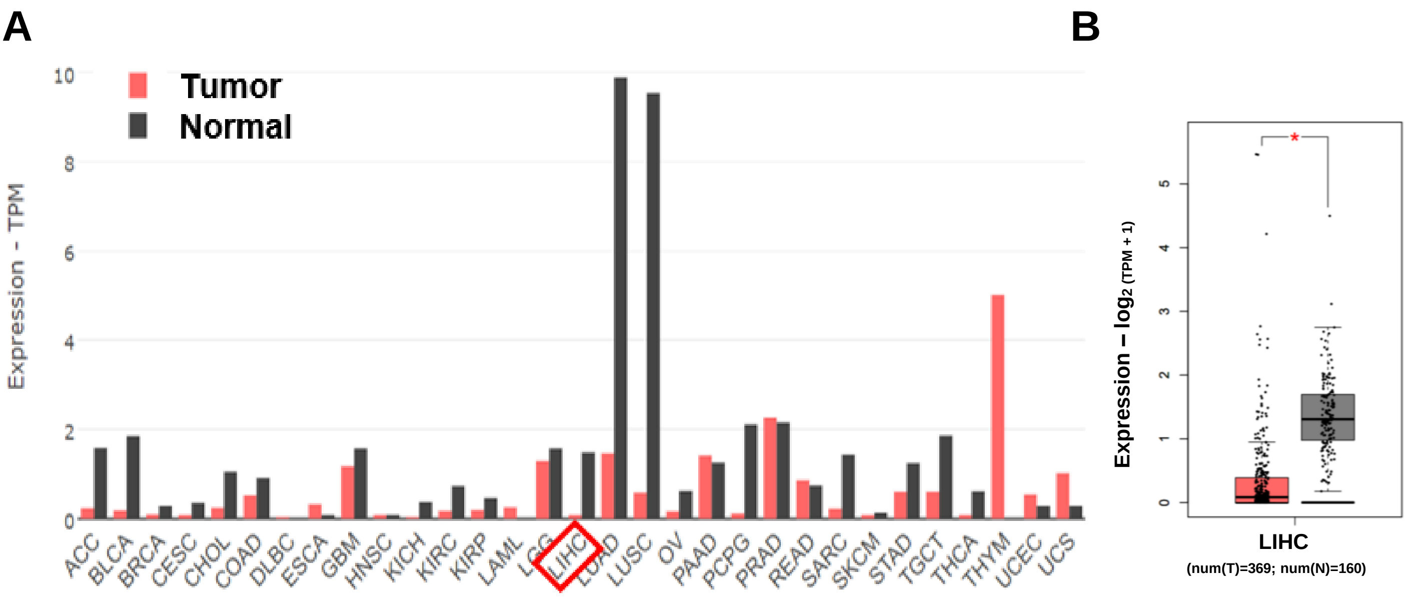

Figure S3. CircFAM114A2 was downregulated in liver cancer tissues. (A) Relative expression levels of HHIP mRNA among different tumor tissues. **(B)** Relative expression levels of HHIP in live cancer tissues.
