# Supplementary material for: CircFAM114A2 inhibits the progression of hepatocellular carcinoma via miR‐630/HHIP axis
Source: Cancer Med. 2023 Apr 11;12(11):12553–68. doi: 10.1002/cam4.5894 (PMC10278467; doi:10.1002/cam4.5894)
Supplement: Supplementary file 2 — Table S1. [file CAM4-12-12553-s001.doc]

**Supplementary Table 1**

| **Table S1. Nucleic acid sequence information of the siRNA and PCR primer** | | |
| --- | --- | --- |
| **siRNA** | **Sequence** | |
|  | **sense** | **antisense** |
| CircFAM114A2 siRNA1 | GCACACUGAUGUUCUAAUCTT | GAUUAGAACAUCAGUGUGCTT |
| CircFAM114A2 siRNA2 | AGAGCACAC UGAUGUUCUATT | UAGAACAUCAGUGUGCUCUTT |
| **Fish probe** | **Sequence** | |
| CircFAM114A2 | ACATGATTAGAACATCAGTGTGCTCTGAACAGCAGT | |
| **RAP probe** | **Sequence** | |
| CircFAM114A2 RAP1 | TCTTTATCTGACATGATTAGAACATCAGTGTGCTCTGAACAGCAGTAGAGATGGTA | |
| CircFAM114A2 RAP2 | ATCTTTATCTGACATGATTAGAACATCAGTGTGCTCTGAACAGCAGTAGAGATGGTAG | |
| CircFAM114A2 RAP3 | CATCTTTATCTGACATGATTAGAACATCAGTGTGCTCTGAACAGCAGTAGAGATGGTAGA | |
| **PCR primer** | **Sequence** | |
|  | **Forward** | **Reverse** |
| CircFAM114A2 | AGAACATCAGTGTGCTCTGAAC | AGCAGGAGAGACAAATGCCAA |
| FAM114A2 | CTCGGAAAAGACCAGAGACCA | GAGGAGAGTATGGACTTGCCC |
| β-actin | GCACAGAGCCTCGCCTT | GTTGTCGACGACGAGCG |
| GAPDH | ATCAATGGAAATCCCATCACCA | GACTCCACGACGTACTCAGCG |
| U6 | GGAACGATACAGAGAAGATTAGC | TGGAACGCTTCACGAATTTGCG |
| CircRBM6 | TTCTGATCAAACACCAGCAGC | TAGGTGGAGGGGGACTCTCT |
| CircSLX4IP | GGAAGTTCGCAAACAGCACA | TGTTTGAACCTTGTGGCAAGA |
| CircRPS6KC1 | GAAGCTCACTCAGATTCCCTCA | GAGGAAACAACTCTGAATGTCGG |
| CircMED13 | CATCAAAGAGGGCCTGGATCT | AACGGGTCCAGATTTCCTGTC |
| CircILKAP | AGCCTTAAGCCTCAGCAAAGA | ACCCGAGTACTGACGTTTCC |
| CircERC1 | AGCTGAAGAAGGAACGAGCC | ACCATATCTGGTTTTCCTCCTGT |
| CircMANBA | TCCTACCCAGGGAATCTGGAA | GGTCCAGTTATCCAAAGAGACCC |
| CircVWA8 | GCCTGCCTTTCCAGGATATGA | AGCATTCACAAGGGGTGAGG |
| CircGON4L | CTGGGCCAGTTACCCAAGAA | TGTTCCATTCTCCTTCTTCCTTGA |
| CircZSWIM6 | CAGTGCACCACACAGAAGTT | TGCAAACAGTTTCTGGCTCC |
| CircKANSL1L | TGTGGCAGTGTTTCCAATACTT | TTTGCTGTTGCCTCCCTCAG |
| HHIP | TCTCAAAGCCTGTTCCACTCA | GCCTCGGCAAGTGTAAAAGAA |
| GRIA2 | CACCCCACATCGACAATTTGG | GACGTGGAGTGTTCCGCAA |
| LSAMP | AGAGTTCAGCCGGATCGGAA | CGTGCCTCGGTTAAAATCCAC |
| ZBTB4 | GAGCGTTCCTACGTGACCC | CTTTCTCACAATAGCGGCAGG |
| PPP2CA | CAAAAGAATCCAACGTGCAAGAG | CGTTCACGGTAACGAACCTT |
| NCKAP1 | TTGTACCCCATAGCAAGTCTCT | GGGCATTTCTCCACTGGTCAG |
| TGIF1 | GGGATTGGCTGTATGAGCACC | GGCGGGAAATTGTGAACTGA |
